# Bacterial nanocellulose: biosynthesis, structural properties and vascular tissue engineering applications

**DOI:** 10.1007/s10856-026-07098-z

**Published:** 2026-06-20

**Authors:** Saeed Sharifpoor Kermani, Jens Wippermann, Priya Veluswamy, Max Wacker

**Affiliations:** Department of Cardiac and Thoracic Surgery, Otto von Guericke University Magdeburg, Magdeburg, Germany

## Abstract

Coronary artery bypass grafting (CABG) via autologous vein or artery is still the gold standard surgery operative revascularization. However autologous vein/artery may represent some limitations like varicose veins or unavailability of veins due to reoperations especially in older patients. consequently, the research focus directed towards identification and characterization of biocompatible alternative material. Bacterial nanocellulose (BNC) as a natural material has gained substantial recognition in the research field of tissue engineering due to it exceptional attributes, including biocompatibility, enhanced mechanical properties, non-toxicity and unique physicochemical properties. Nevertheless, BNC is associated with some restrictions in endothelial cell adhesion and mimicking the physiochemical and mechanical properties of autologous vein as a promising alternative. To address these challenges, future research must focus on the development of BNC with controlled surface and mechanical characteristic. This review aims to address diverse aspect of controlling BNC production and methodologies in order to optimize the mechanical and physiochemical properties of grafts as an alternative in cardiovascular tissue engineering. Created with BioRender.com.

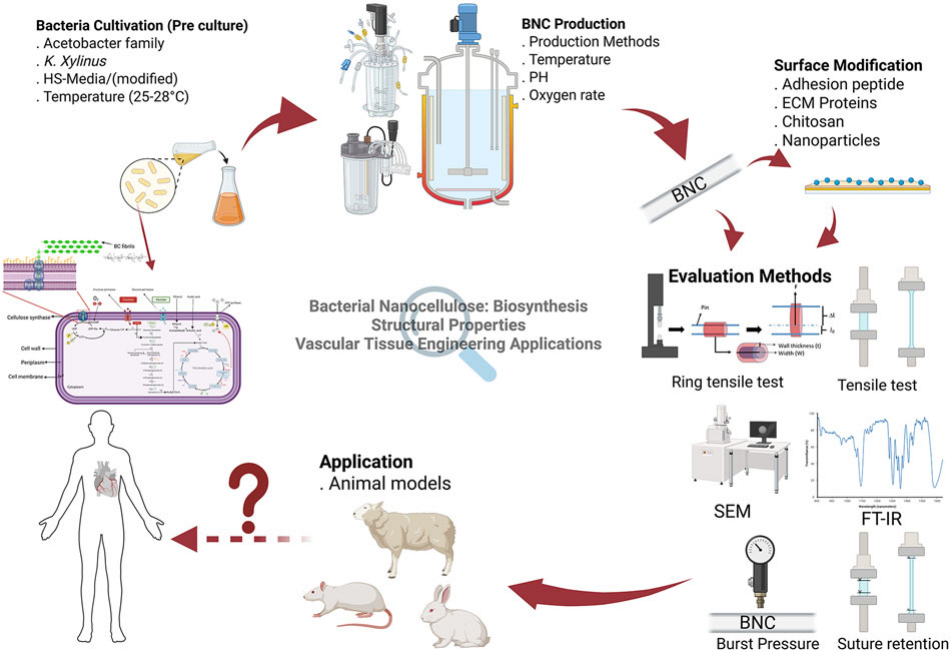

## Introduction

Cardiovascular diseases (CVDs) are the major cause of death, accounting for up to 32% of all deaths across the European Union (EU) in 2021. In 2022, CVDs were the most common cause of death in Germany, attributing to 208,578 deaths [1]. Among CVDs, coronary artery disease (CAD) is considered to be one of the main causes of mortality. More specifically, the age-standardized incidence of CAD in Germany decreased from 0.8% in 2012 to 0.7% in 2022. Despite this decline, approximately 6.9% of the German population (7.3 million individuals) are living with CAD according to the published report in2023 [1], indicating that the overall prevalence of CAD remains significant [2, 3]. Consequently, the treatment of severe CAD with coronary artery bypass grafting (CABG) is one of the most frequent heart surgeries in the western world. About 37,000 CABG procedures were performed in Germany in 2023 [4]. CABG is regarded as the gold standard for operative revascularization, where an autologous vein or artery from the same patient is implanted as conduits to guide the blood around the stenosed vessel section. Synthetic grafts, such as expanded polytetrafluoroethylene (ePTFE, e.g., Gore-Tex®) and polyethylene terephthalate (PET, i.e., Dacron), are not used in CABG due to their low patency rates but may be applied in peripheral vascular surgery when autologous grafts are unavailable [5, 6]. However, synthetic grafts demonstrated limitations such as poor endothelialization and high occlusion rates [7, 8]. Though autologous grafts are considered to be the gold standard for CABG, there exist some limitations including the potential of varicose veins or unavailability of veins due to reoperations especially in older patients. To address these challenges and to overcome these limitations, recent researches has focused on developing a better alternative biocompatible material. Being a natural material, bacterial nanocellulose (BNC) has made immense recognitions in the research field of tissue engineering due to its unique meritorious attributes such as biocompatibility and non-toxicity with enhanced mechanical properties, which might emerge as a promising alternative candidate in comparison to other described vascular grafting materials [9]. However, BNC may have limitations in endothelial cell adhesion, which is crucial for vascular graft success, and the production of grafts with surfaces and mechanical characteristics that mimic autologous vessels is a topic that is still being researched. This review focuses specifically on the requirements of cardiovascular tissue engineering and the development of small-diameter vascular grafts. It summarizes current knowledge on the control of BNC biosynthesis, methods for structural characterization, strategies for surface modification, and biomechanical evaluations, with particular emphasis on their relevance to vascular graft performance. Thus, this review aims to provide an application-oriented perspective on how the properties of BNC can be adapted to meet the mechanical and biological requirements of vascular grafts.

## BNC production

Controlled bacterial cellulose production is achieved by regulating several factors for obtaining better BNC production. These factors include (i) strain/type of cellulose-producing bacteria, (ii) composition of bacterial culture medium, (iii) gas conditions, (iv) pH and (v) temperature. By modifying the aforementioned conditions, researchers can precisely fine-tune the environment for BNC production with required physical, chemical and mechanical properties.

### Bacteria and medium

The first production of nanoscale cellulose through fermentation by *Bacterium aceti* in presence of oxygen and glucose was reported in 1886 [10]. These bacteria naturally produce BNC as an envelope that protects them from harsh environmental conditions [11, 12].

One of the most frequently used BNC-producing bacteria is *Komagataeibacter*, which belongs to the family of *Acetobacteraceae*. Gullo et al. showed that *Komagateibacter* x*ylinus* species has the highest rate of cellulose production among other 34 species [13]. *Komagataeibacter sp*. is characterized as Gram-negative, aerobic and nonpathogenic and produces nanofibril cellulose using sugars like glucose and fructose [14]. Moreover, comparative genomic analysis has confirmed that *Komagataeibacter* species are considered to be the most efficient bacterial cellulose producers due to their well-developed operons for cellulose biosynthesis [15–17]. These bacteria synthesize extracellular polysaccharides under suitable environmental conditions in the presence of carbon sources like glucose, fructose or ethanol, involving various intracellular pathways [18]. Depending on the carbon source, these substrates are first converted into glucose-6-phosphate, which serves as the central precursor for cellulose biosynthesis. For example, fructose can be converted into fructose-6-phosphate and subsequently into glucose-6-phosphate before entering the pathway. These mechanisms include the conversion of a carbon source (intracellularly) to a crystalized cellulose (polysaccharide, extracellular), which is then secreted through the cell membrane to the environment. Intracellular conversion of a glucose as an example, into cellulose includes four following steps:

1. Glucose phosphorylation via glucokinase into glucose-6-phosphate
2. Glucose-6-phosphate isomerization via phosphoglucomutase into glucose-1-phosphate (linear glucan chain)
3. Uridine diphosphate glucose (UDP-glucose) synthesis from glucose-1-phosphate via UDP-glucose pyrophosphorylase
4. Polymerization of UDP-glucose into cellulose via cellulose synthesis complexes (from cell membrane to the environment).

The bacterial cellulose synthesis complexes (bacterial cellulose synthase, Bcs) consist of A, B, C and D subunits. While BcsA and BcsB participate in BNC synthesis, BcsC and BcsD are responsible in glucan transport and loading on the surface of the cell [18–21].

**Fig. 1:**
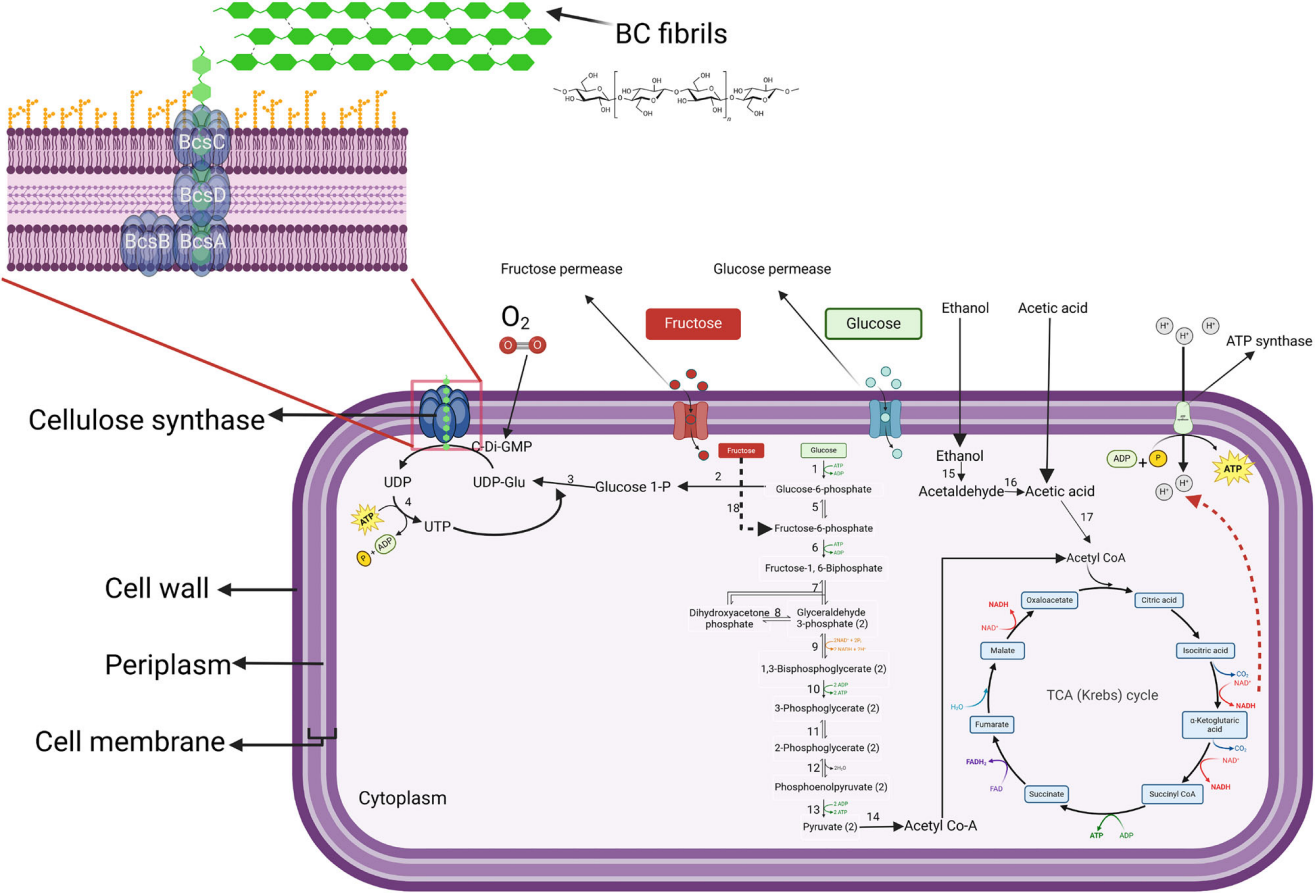
Schematic representation of pathways involved in BNC biosynthesis inside K. xylinus and subsequent assembly of cellulose molecules into nanofibrils: (1) Glucokinase-ATP, (2) Phosphoglucomutase, (3) UDP-glucose pyrophosphorylase, (4) Nucleoside diphosphate kinase (NDK), (5) Phosphoglucoisomerase, (6) Fructokinase ATP, (7) Aldolase, (8) Triosephosphate isomerase, (9) Glyceraldehyde-3-phosphate dehydrogenase (GAPDH), (10) Phospho-glycerate kinase (PGL), (11) Phosphoglycerate mutase, (12) Enolase, (13) Pyruvate biphosphate kinase, (14) Pyruvate dehydrogenase, (15) Alcohol Dehydrogenase (ADH), (16) Aldehyde Dehydrogenase (ALDH), (17) Ace-tyl-CoA Synthetase (ACS). Created with BioRender.com.

In general, the UDP-glucose-pyrophosphorylase plays a very important role in cellulose production in cytoplasm of bacteria. Of note, the activity of UDP is much higher in cellulose-producing bacteria compared to those without this ability. This enzyme initiates the production as well as transport of cellulose fibrils from the cytoplasm to the outside of the membrane [22, 23]. The cellulose biosynthesis associated pathways inside the bacteria *K.xylinus* is shown in Fig. 1, including (i) the Krebs cycle, (ii) pentose phosphate pathway (PP), (iii) gluconeogenesis (GNG) and (iv) Embden-Meyerhof-Parnas pathway (EMP) [18, 19].

The production of bacterial cellulose can be manipulated using different substrates. This implies that one of the aspects for controlling the BNC production can be achieved by adding appropriate substrates (as source) into the culture media. In BNC-producing bacteria, metabolic pathways such as GNG and PP play crucial roles in supporting the process of BNC production. The GNG pathway is responsible for converting acetate into pyruvate, which is then used as a building block for cellulose, whereas the PP cycle supplies important molecules like reduced nicotinamide adenine dinucleotide (NADH), which provides energy for biosynthesis and sugar phosphates that support cell growth and cellulose formation. The most commonly used medium to culture *Komagateibacter sp*. for BNC production is Hestrin-Schramm medium (HS medium). It contains 2% glucose (as main carbon source), 0.3% peptone, 0.5% yeast extract, 0.27% dibasic sodium phosphate and 0.114% citric acid [24]. Despite production methods, the concentration and type of carbon sources in the media may have a substantial outcome on the physical properties of BNC, such as yield, wall thickness and porosity [25, 26]. This was proven when different carbon sources were investigated for BNC production, where glucose, fructose and glycerol were found to change both BNC yield and production rate, which were demonstrated using different *Komagataeibacter* strains [9, 27]. Such differences in carbon source could bring changes in the BNC morphologic structure and crystal size [20, 28]. Table 1 shows the crystal properties of BNC synthesized from *Komagataeibacter xylinus* (ATCC 23770) using various carbon sources.

**Table 1:** Crystal properties of BNC from different carbon sources (adopted from [28])

Carbon sources	Crystallinity index (%)	Crystallite size [nm]			
		L (101)	L (10i)	L (002)	L (040)
Galactose	78	4.73	3.40	5.58	2.39
Xylose	77	3.95	4.17	4.53	4.84
Mannose	76	4.24	4.28	4.45	4.98
Maltose	71	4.14	4.37	4.76	5.08
Arabinose	67	5.07	5.13	5.32	5.96
Glucose	61	4.27	4.32	4.49	4.99
Sucrose	61	5.17	5.24	5.43	6.04

The most commonly used carbon sources for BNC production are glucose, fructose and sometimes alcohol (glycerol). Of note, fructose and glycerol might affect the porosity, and glucose might impact the structural density of the produced BNC [9]. Also, these components might influence the BNC production rate. Molina-Ramírez et al. reported that the 2% v/v glucose has the highest BNC yield (2.80 g/L) compared to sucrose (1.68 g/L) and fructose (0.38 g/L) [29].

However, the BNC-producing bacteria are not able to completely convert the prevailing carbon source into BNC. For example, the metabolic flux analysis of the bacteria *K. xylinus* revealed that it can only convert about 19-24% of the carbon into a BNC when glucose is used as a carbon source. The remaining carbon is gradually oxidized to gluconic acid (as a by-product), which could reduce the BNC yield due to less availability of glucose for further BNC production (termed as carbon flux diversion). These converted gluconic acids reduce the pH of the culture medium due to their acidic nature. This acidification reduces the activity of enzymes such as cellulose synthase, which are essential for the production of nanocellulose. Therefore, it affects cellulose production. Additionally, the accumulation of gluconic acid increases osmotic pressure outside the cells, causing osmotic stress that leads to cellular dehydration and disrupted nutrient uptake, which together further inhibit BNC production [30–32].

It has been reported that the addition of specific supplements such as organic acids, vitamin C and alcohol can further improve the BNC yield. For instance, supplements like lactic acid and ethanol could promote BNC yield in *K. xylinus* [33] and *Gluconacetobacter kombuchae* [34].

At the molecular level, ethanol improves the transportation and phosphorylation of glucose and subsequent synthesis of UDP-glucose. Simultaneously, ethanol suppresses the expression of key genes involved in glycolysis and gluconate production, such as Glucose dehydrogenase (gcd), Gluconate (or glucose/ gluconate) transporter genes (gntT1 and gntT2), Glyceralde-hyde-3-phosphate dehydrogenase (gap), Phosphoglycerate kinase (pgk), Pyruvate, phosphate dikinase (ppdk), and Glucose-6-phosphate dehydrogenase/ Zwischenferment (zwf1), which reduces the conversion of glucose to gluconate and subsequent production of gluconate-6-phosphate, shifting glucose metabolism towards cellulose biosynthesis [35, 36], while lactic acid is responsible for boosting the carbon activity in TCA cycle leading to improved cell growth [37], making these two supplements (ethanol and lactic acid) promising candidates to enhance the BNC yield using *K. xylinus*. Henceforth, the carbon source conversion efficiency and the formation of by-products such as gluconic acid are a major limitation in the production process of BNC. This limitation may be mitigated by using alternative carbon by using alternative carbon sources (e.g., glycerol or fructose), optimizing the culture medium with pH buffers or additives (e.g., ethanol, lactic acid, vitamin C), or genetic modifications that inhibit gluconic acid metabolism and promote cellulose biosynthesis.

### Oxygen

One of the main characteristics of *Komagataeibacter sp*. is their oxygen-dependent cellulose production. Variations in oxygen availability and the partial pressure of oxygen (pO_2_) strongly affect bacterial growth, metabolism and BNC synthesis. In contrast, strictly anaerobic conditions inhibit cellulose formation. By regulating the partial pressure of oxygen (pO_2_) in the bacterial microenvironment, the required cell growth and controlled cellulose productivity can be easily achieved [19]. Hülsmann et al. reported that by decreasing the supply of pO_2_ to 4%, it could be possible to enhance bacterial growth in static condition without BNC production, which helps to control the cell numbers before initiating the BNC production [38].

### pH and temperature

pH and temperature are two key parameters that directly affect the BNC production. pH variations, especially a decrease in pH, may have a detrimental effect on the enzyme activity of crucial enzymes such as cellulose synthase, whose inhibition reduces bacterial growth and contributes towards a low BNC yield [39]. Since the K. xylinus belongs to the family of *Acetobacteraceae*, it tends to produce BNC at low pH. Apart by-product accumulation (e.g., gluconic acid) can also be involved in lowering the pH. The optimal pH for *K. xylinus* cultivation is 5.5-6.0, whereas for other *K.sp*. pH ranges between 4.5 and 7.5 [39, 40].

Likewise, temperature is another major parameter that influences the production of BNC, where temperature ranging from 25 °C to 30 °C was found to the best for *Komagataeibacter species* to synthesize BNC [14, 41, 42]. Deviations from this temperature range can negatively affect the intracellular metabolic processes responsible for cellulose biosynthesis. Temperature alterations affect the action of cellulose synthase enzymes and induce stress responses such as the activation of heat-shock proteins, which may divert cellular energy from metabolic pathways involved in BNC synthesis. Park, Myung Soo, et al. investigated the impact of various temperatures (20 °C, 25 °C, 30 °C, and 37 °C) on *Komagataeibacter sp*. (SFCB22-18) BNC synthesis. The result indicated that the BNC yield was maximum at 30 °C, with a significant decline in yield when the temperature was decreased or increased [43]. This signifies the importance of temperature on bacterial metabolic processes essential for BNC production. However, the ideal temperature for *K. xylinus* is 28 °C. Temperatures above 30 °C can cause denaturation of essential proteins utilized in bacterial nanocellulose synthesis, such as cellulose synthase and other regulatory enzymes, while low temperature (< 20 °C) hampers the enzymes and the metabolism [14, 39, 44]. Thus, pH and temperature conditions are crucial factors for efficient BNC production, since variations from the optimal range (pH 5.5–6.0 and 25–30 °C) can affect the activity of important enzymes such as cellulose synthase, which may reduce the yield. These limitations can be addressed by keeping the pH stable using suitable buffer solutions or continuous pH regulation, by precisely controlling the fermentation temperature or keeping it constant within the optimal range using automated bioreactor systems in order to maximize enzyme activity and cell metabolism.

Besides environmental parameters such as pH and temperature, bacterial regulatory mechanisms can also influence BNC biosynthesis and the properties of the material produced.

R. E. Jabbour et al. investigated the effect of quorum-sensing molecules (N-acyl-homoserine lactones, AHLs) on the quality of bacterial nanocellulose. The AHLs are vital quorum-sensing (QS) molecules that enable bacteria to regulate their growth behavior through the overexpression of the LuxR protein (QS-TF: Quorum Sensing Transcription Factor), which leads to an increase in nanocellulose production [45, 46].

Bacterial nanocellulose biofilms were produced using a *Komagataeibacter xylinus* strain 10245, cultured in HS medium, in 50 mL conical tubes at 27 °C for 3−7 days. Three types of AHLs (N-decanoyl-DL-homoserine lactone (DHL), N-dodecanoyl-L-homoserine lactone (DDHL), and N-(3-oxododecanoyl)-L-homoserine lactone (ODDHL)) were dissolved in ethyl acetate and added to the bacterial growth media at a concentration of 5 mg/L. Upon post processing, the BNC pellicles were dried at 30 °C for 24 h, and the BNC was extensively characterized. The results showed that the DHL-BNC sample had an overall more uniform structure compared to the ODDHL-BNC. Furthermore, the utilization of DDHL in the growth media may result in amorphous fibrils compared to DHL or ODDHL [46].

After BNC production, the produced BNC are not immediately suitable for biomedical application, since the bacterial cells and extracellular DNA (eDNA), medium components and metabolic byproducts are embedded within the structure of the fermented BNC graft. Moreover, Gram-negative bacteria such as *Komagataeibacter* species may release endotoxin, which represents a safety risk for their biomedical application in vivo. Thus, purification of BNC is a crucial step to ensure biocompatibility and clinical suitability [9, 47, 48]. Various purification strategies for bacterial nanocellulose have been described in the literature. Among these approaches, alkaline treatment represents the most commonly applied method. In this method, alkaline solutions such as sodium hydroxide (NaOH) are applied to remove bacterial cells and residual impurities, followed by washing with distilled water until neutral pH is achieved [9, 48–50].

### BNC properties

Bacterial nanocellulose is widely used in drug delivery, wound healing, tissue engineering, and biomedical and pharmaceutical industries due to its unique morphological and physical properties such as high tensile strength, water-holding, flexibility and elasticity [9, 51–53]. The application of BNC in these different fields largely depends on its structural, morphological, and chemical characteristics, which can vary based on the type of bacteria, fermentation conditions, and processing methods. Therefore, a systematic understanding of BNC’s properties is essential for selecting or modifying it for specific applications. Such BNC properties include fiber diameter, crystallinity, porosity, surface area, thermal stability, and mechanical strength. Various methods exist to investigate the physical and chemical properties of BNC, which are described in this section. Importantly, single-parameter analysis may not provide a comprehensive overview of BNC structural properties. But, a combination of BNC evaluation methods is needed to obtain a better understanding of its characteristics [54, 55]

#### Structure properties

In order to better comprehend the BNC structure, it is advisable to first understand the classification, origin, and properties of cellulose, thereby enabling the appropriate selection of methods needed to analyze the structural properties.

Cellulose consists of polysaccharides made of D-Glucose units (D-anhydroglucopyranose), linked via β 1 → 4 glycosidic bonds and contains a high amount of hydroxyl groups [55, 56]. These linear units (D-Glucose) are able to connect each other via hydrogen bonding (intra- and inter-molecular bonds), resulting in crystallization of cellulose chains to form a parallel insoluble microfibril structure. This microfibril structure consists of two regions (crystalline and amorphous regions) which give the cellulose a high strength, biocompatibility and stiffness. The presence of hydroxyl groups in each monomeric unit of cellulose plays a pivotal role in determining its properties, including hydrophilicity, chirality, reactivity, and biodegradability. Furthermore, these hydroxyl groups can participate in various chemical modifications such as etherification, hydroxypropylation, carboxymethylation, etc., leading to cellulose derivatives [54–56].

At the nanoscale, the cellulose units link together with a certain dimension to form nanocellulose, which is categorized into three groups based on their resources:

1. Bacterial nanocellulose (BNC) using bottom-up methods, where bacteria produce cellulose at the nanoscale directly.
2. Cellulose nanofibers (CNFs) using top-down approach, where mechanical or chemical processes break down larger cellulose structures into nanoscale fibers.
3. Cellulose nanocrystals (CNCs) using top-down methods, typically through acid hydrolysis, which isolates the crystalline regions of cellulose.

**Fig. 2:**
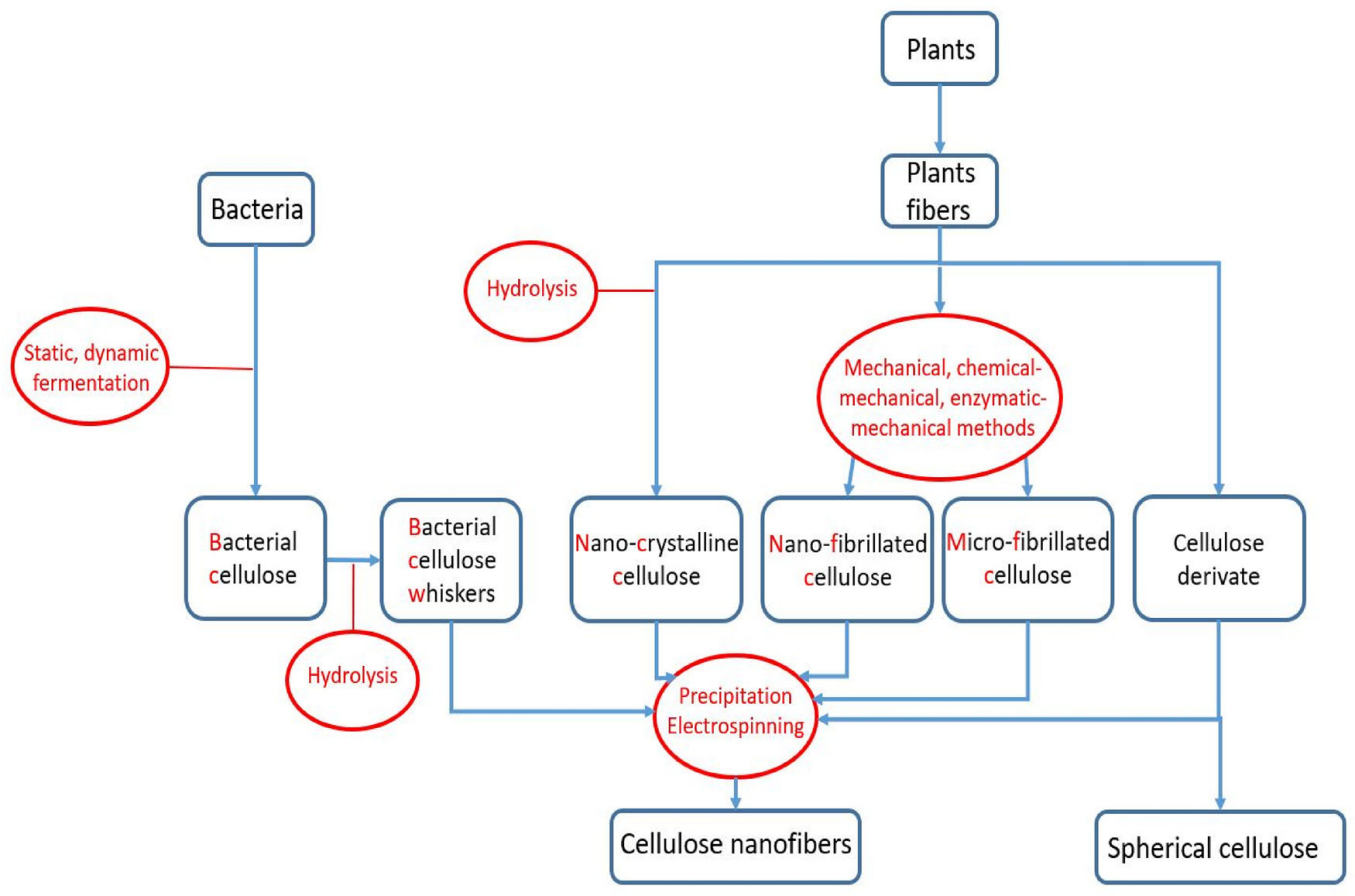
Sources, preparation, and classification of nanocellulose [adopted from [48, 49]]

CNFs and CNCs are mainly synthesized from plant fiber whereas BNC is produced from microorganisms, particularly species of the genus *Komagataeibacter* (formerly classified as Gluconacetobacter) [17, 55, 57, 58]. Figure 2 shows the sources, preparation, and classification of nanocellulose [59, 60].

Furthermore, several properties of CNCs, NFCs, and BNCs such as dimension, mechanical properties, and crystallinity are detailed in Table 2.

**Table 2:** Physical and chemical properties of various type of nanocellulose (adapted from [45])

Type of cellulose	Dimension	Crystallinity	Hydrophilic/ Hydrophobic nature
CNC	Diameter: 2-20 nm Length: 100-500 nm or several micrometer	54-88%	Highly hydrophilic
CNF	Diameter: 1-100 nm Length: 500-2000 nm	45-80%	Hydrophilic
BNC	Diameter: 25-80 nm Length: several millimeter	45-89%	Highly hydrophilic

The microfibril structure and high crystallinity of bacterial nanocellulose (BNC) give it excellent mechanical properties, making it a promising biopolymer for applications in cardiovascular tissue engineering [49, 55].

Recently, different methods have been applied to evaluate the physical and chemical properties of BNC. However, not all of these methods are equally relevant for the evaluation of BNC as a vascular graft material. The evaluation of vascular grafts should follow DIN EN ISO 7198:2017, which define the essential requirements for vascular prostheses. These requirements include mechanical testing (tensile properties, burst pressure, suture retention, and compliance) as well as structural characterization (wall thickness and material structure). In addition, biocompatibility, including hemocompatibility and thrombogenicity are characterized according to ISO 10993. The standard ISO 7198:2017 does not prescribe the specific analytical technique for surface characterization. Thus, the selection of methods should be based on the biological relevance of the graft. In this context, scanning electron microscopy (SEM) can be considered essential for the evaluation the fibril structure, pore architecture and surface morphology, while X-ray photoelectron spectroscopy (XPS) and Fourier-transform infrared spectroscopy (FT-IR) provide key information on the chemistry and functional groups. Atomic force microscopy (AFM) may be applied as a complementary method for roughness and topography of the surface, whereas energy-dispersive X-ray (EDX) provides supportive elemental analysis with limited surface sensitivity compared to XPS.

In contrast, X-ray diffraction (XRD) mainly provides information on crystallinity and internal structure and is therefore less relevant for surface related biological interaction and can be considered an auxiliary technique with partial redundancy.

For cardiovascular applications, morphological characterization techniques such as SEM and AFM remain important for evaluating the fibril structure, pore architecture, and surface morphology and roughness, which influence endothelialization.

Some of the crucial techniques involves tensile and specially ring tensile test (for tubular shape graft), burst pressure and suture retention test (In surgical aspect), which are described in Section 4.

##### Scanning electron microscopy (SEM)

SEM is a widely used method which gives insight into the topography (distribution of features or components on the surface of a sample), the morphology (shape or structure of a sample), the composition (what a sample is made of) and the crystallographic and structural nature. In SEM, a focused electron beam is directed at the sample surface, where it interacts with the atoms of the sample and generates signals. These signals consist of secondary electrons, backscattered electrons, and characteristic X-rays. By detecting and analyzing these signals, SEM provides detailed information about the two-dimensional surface topography of the sample, including the size and shape, making it an important tool for investigating the fiber orientation and size, pore size and surface coating of BNCs (if the BNC is coated). The samples require sample processing such as dehydration, fixation and metallization [61–65]. Due to the high water content of BNC, it must be dried to avoid gassing under vacuum, which results in poor image quality and can possibly damage the apparatus [66, 67]. To overcome this problem, the BNC samples should be dried. This can be achieved by using (i) climate chamber at 23 °C, (ii) an oven at 100 °C and/or (iii) freeze-drying. Furthermore, the samples need surface sputtering with gold or any equivalent conductive material. This is because BNC is a non-conductive material, which reduces charging, improves image quality and enhances secondary electron emission, resulting in optimal imaging conditions in SEM [64, 65, 68–70]. It should be noted that any kind of drying will change the morphology of the BNC fibers composition. To better maintain physiologically relevant structures, techniques such as freeze-drying or cryogenic preparation (cryo-SEM) are increasingly being applied to provide a more accurate representation of the native hydrated fibrillar network of BNC. In addition, the mechanical characterization of BNC scaffolds is typically performed under hydrated, providing data that are a more accurate representation of their behavior under physiological conditions.

Cryo-SEM is another specialized form of scanning electron microscopy (SEM) that is applied to examine samples at cryogenic temperatures with liquid nitrogen. This method involves rapid freezing of the sample to preserve its native state and prevent deformations that occur when the sample is dried or freeze-dried. The merits of Cryo-SEM include preservation of the native structure, as it maintains the natural, hydrated state of biological and nonconductive samples like BNC in freezing state, and preserving their morphology and structural integrity as close as possible to the original state. Reports on the use of Cryo-SEM for analyzing surface-modified BNC and the resulting cell–cell interactions are still scarce; however, this technique holds significant potential for studies involving surface-modified BNC. This method allows the researchers to observe how the surface modification affects cell behavior [71]. In parallel. Cryo-SEM has the advantage of reducing sample artefacts, which improves the imaging quality and accuracy [72–74].

##### Energy dispersive X-ray (EDX) and X-ray photoelectron spectroscopy (XPS)

EDX is a technique to determine the chemical composition within the near-surface regions of materials, such as BNC. This method involves the directed application of a high-energy beam onto the sample’s surface, resulting in the emission of characteristic X-rays, enabling the imaging and quantification of elemental concentrations at the nanoscale [75–77].

Since BNC consists of carbon, oxygen and hydrogen atoms, peaks corresponding to carbon and oxygen will be observed. However, hydrogen, due to its low atomic number, cannot be detected by these techniques. The processing of the samples is similar to the SEM methodology. They have to be dehydrated, fixed and metallized, which leads to a high image quality and a better recognition of the chemical composition [65, 77].

XPS is another surface analytical method, like EDX, that provides information about elemental composition of the material. Compared to EDX, this method can detect all elements except hydrogen (H) and helium (He), and it is particularly sensitive to lighter elements like carbon (C), Oxygen (O) and Nitrogen (N) with a lower ordinal number <10, making it suitable for detection of the elemental composition of BNC at the surface. The EDX, however, is not able to differentiate between different chemical states and detect light elements with a low ordinal number <10, since it has lower energy resolution [78–81].

##### Fourier transform infrared spectroscopy (FTIR)

FTIR is one of the methods that characterizes the surface chemical of BNC [82, 83]. The technique is based on the absorption of infrared light. This method offers several advantages, including rapid analysis, reproducibility, non-destructiveness (requires no alteration or disruption of the sample’s structure), and the capacity to detect a wide range of chemical functional groups in various materials, allowing for comprehensive identification of molecular structures and surface modifications [84].

This method has several limitations, such as limited information on crystallinity or morphology of BNC, which is critical for understanding its mechanical properties. Since BNC contains a large amount of water, the absorption bonds of water may mask some of the peaks of interest. As drying technique is the main step for sample preparation, it could potentially alter the BNC's original state. Andree, V. et al. reported that the BNC samples which undergoes oven-drying performed poorly compared to freeze-drying. During drying, intermolecular hydrogen bonds between cellulose chains could reformed, affect the crystallinity, leading to collapsing the network structure, which resulted in to shift FTIR signals (especially OH and CH bands), making spectra unrepresentative for wet-state of BNC [68, 85–88].

##### X-ray diffraction (XRD)

XRD is an important non-destructive analytical technique designed to provide detailed information on the chemical composition, crystal structure, crystal orientation, crystallite size, lattice strain, preferred orientation and film thickness. This versatile method is applied to a wide range of samples, including powders, bulk materials, thin films and nanomaterials, and allows comprehensive characterization of nano-structured materials like BNC. The structural properties can be achieved by irradiating them with X-rays, electromagnetic waves with a wavelength of around 0.1 nm. Electron and X-ray interactions within the sample generate scattering that forms diffraction patterns depending on the beam direction and sample orientation. The crystalline solids produce clear diffraction peaks, while amorphous polymers produce broad patterns that do include useful information about the local atomic structure, e.g., bond length and morphology. XRD techniques are generally divided into wide-angle X-ray scattering (WAXS) for analyzing small structures (~1 nm) and small-angle X-ray scattering (SAXS) for observing large features from 1 nm to 400 nm [89–93].

This method is capable of investigating the crystallinity index (CrI, %) using Segal’s equation and crystallite size (L) with Scherrer’s formula [94, 95]:

$${CrI}\left( \% \right)=\frac{{I}_{200}-\,{I}_{{am}}}{{I}_{200}}\times 100$$

$$L=\,\frac{K\lambda }{\beta \cos \theta }$$

Here, I_200_ is the intensity value for the crystalline cellulose, *I*_am_ the intensity value for the amorphous cellulose, *K* = 0.89, the shape factor for Scherrer’s constant, *λ* the x-ray wavelength, *β* in radians, the full width at half-maximum (FWHM) and *θ*, the Bragg angle. Despite being a valuable technique for evaluating the crystalline structure and phase composition of bacterial nanocellulose, XRD has its limitations in terms of sample preparation (grinding), information on the amorphous fraction and possible overlapping peaks. Hence, complementary techniques are required for a comprehensive structural analysis of BNC. Moreover, freeze drying seems to be the most effective option for the sample preparation of BNC, since grinding and pulverization is challenging due to the water content and structure of BNC [94–98].

## Surface modification

In recent years, various studies have investigated the potential of BNC-made small-caliber vascular grafts as an alternative to autologous vessels or synthetic grafts, such as e.g. PTFE. The unique properties of BNC grafts, such as integrating with surrounding host tissues or arteries, biocompatibility, thermal stability, mechanical flexibility, and hemocompatibility, render them favorable in comparison to other synthetic grafts. Despite these unique properties, the BNC is not yet capable of fully performing the characteristics of blood veins due to different factors. The most important factor for long-term patency of small-caliber vascular grafts is considered a throughout endothelialization of the graft [99–102].

Endothelialization enhances the hemo-and cytocompatibilities of the BNC vascular prosthesis by increasing cell attachment and proliferation because of the innate anticoagulant properties of endothelial cells. This could be achieved by either in vitro pre-seeding of BNC with endothelial cells or direct in vivo deposition of circulating endothelial progenitor cells (EPCs), or migration of vascular endothelial cells from native vessel onto the implanted graft or migration of the vascular endothelial cells from surrounding capillaries onto the graft from its outer porous region. To facilitate the process of endothelialization, different surface modification methods have been investigated, using (i) extracellular matrix proteins (fibronectin or collagen), (ii) adhesion peptides (arginine-glycine-aspartic acid (RGD), (iii) Chimeric proteins containing a cellulose-binding module (CBM) fused to adhesion peptide sequences (RGD or GRGDY), and (iv) metal nanoparticles and anticoagulants like heparin, as summarized in Table 3 [99–103].

**Table 3:** Different surface modification methods of BNC using various components

Class	Component	Cell type	Method	Result/Advantages	Reference
**Adhesion peptides**	Arginine-glycine-aspartic acid (RGD), glycine-arginine-glycine-aspartic acid-serine (GRGDS), glycine-arginine-glycine-aspartic acid-tyrosine	Fibroblasts, HMECs, neuroblasts, and mesenchymal stem cells	Arginine-glycine-aspartic acid (RGD) or its variations like glycine-arginine-glycine-aspartic acid-serine (GRGDS) and glycine-arginine-glycine-aspartic acid-tyrosine linked to BNC through functionalization with cellulose-binding module (CBM)	Enhancing cell growth, proliferation, distribution, and elongation	[102, 103, 149, 150]
**ECM protein**	Fibronectin	VECs and EPCs	Coating of BNC grafts with fibronectin followed by static and dynamic cell seeding	Significant increased cell adhesion and proliferation	[106]
	Gelatine	HUVEC and SMC	Chemically cross-linking gelatin using glutaraldehyde to BNC	Significant increase cell adhesion and proliferation, no significant effect on whole blood clotting time	[151]
	Collagen type I	MSC	1-cyano-4-dimethylamino pyridinium tetrafluoroborate (CDAP) crosslinking	Significantly higher cell proliferation and cell elongation	[152]
**Plasma protein**	Albumin	VECs and EPCs	Coating of BNC grafts with albumin followed by static and dynamic cell seeding	Promoted cell adhesion of VECs only, endothelial functionality was impaired	[106]
**Magnetic force using nanoparticle**	Iron oxide nanoparticles (IONs)	Murine endothelial cells	Forming PEG-IONs by functionization of carboxylated PEG with IONs	Enhanced adhesion and proliferation	[153]
**Anticoagulant agent**	Heparin	HUVECs, and SMCs	Chemically functionalized using 1-ethyl-3-(3-dimethylaminopropyl) carbodiimide/N-hydroxysuccinimide (EDC/NHS) cross-linking	Greater proliferation of HUVECs but lower proliferation of human SMCs	[100]

*HMECs* Human Microvascular Endothelial Cells, *HUVECs* Human Umbilical Vein Endothelial Cells, *VECs* Human saphenous vein endothelial cells, *EPCs* Endothelial progenitor cells, *MSCs* Mesenchymal Stromal Cells, *SMCs* Smooth Muscle Cells

However, the application of these components presents several limitations. For heparin, the covalent bonding technique may alter the conformation of heparin molecules, potentially reducing their ability to interact with antithrombin III, which is essential for anticoagulant function. As a result, the coating efficiency and bioactivity, as well as cell binding ability and anticoagulant activity, may be reduced [102, 104, 105]. Some literature stated that although fibronectin significantly enhances cell adhesion and proliferation, it could also activate platelets and trigger thrombosis [106–108]. Through conjugation of two or more components, it will be possible to enhance their effects on hemo- and cytocompatibilities, but more investigation is still needed. Thus, though small-diameter BNC-based vascular grafts showed excellent biocompatibility, mechanical flexibility, and hemocompatibility, there is a significant challenge in achieving long-term patency due to their limited ability to achieve complete and stable endothelialization. This challenge may be overcome by optimizing surface modification strategies, such as combining multiple bioactive coatings (e.g., heparin with ECM proteins or RGD peptides), improving immobilization methods to enhance molecular bioactivity, and applying enhanced biofunctionalization or dynamic cell seeding techniques to enhance sustained endothelial cell adhesion, proliferation, and antithrombotic performance.

## Biomechanical properties and test methods

The biomechanical requirements for small-diameter vascular grafts are relatively simple: from the mechanical point of view, the goal is to develop grafts with high mechanical strength that simultaneously maintain a physiological compliance. Since natural blood vessels exhibit a viscoelastic behavior, the behavior of the vessel wall depends on both pressure-dependent elastic properties and time-dependent viscous properties. Despite numerous research attempts, it has not yet been possible to develop small-diameter vascular grafts that fully mimic these properties [109, 110].

The key biomechanical parameters for evaluating vascular grafts include tensile strength, compliance, suture strength and burst pressure. These parameters are directly related to the graft’s performance and long-term patency after implantation: A mismatch between the vascular graft and the native vessel may lead to disturbed hemodynamics, endothelial dysfunction and the development of neointimal hyperplasia, which are major causes of graft failure. Thus, systematic evaluation of these biomechanical parameters is essential when assessing biomaterials for vascular graft application.

Tensile strength describes the material’s resistance to mechanical stress; compliance refers to the circumferential elasticity of the graft, burst pressure reflects the graft’s ability to resist physiological and supraphysiological blood pressure; and suture strength determines the mechanical stability of the graft during surgical anastomosis. However, the optimal target values for these parameters do not yet have a consensus. Furthermore, the question of whether autologous vessels such as the internal mammary artery or the saphenous vein should serve as universal reference standards remains unclear, as arterial and venous tissue differ significantly in their mechanical properties [111]. In addition, these parameters are typically determined under controlled in vitro conditions (e.g., uniaxial tensile testing, pressurization systems or suture pull-out tests), which may not fully represent the complex in vivo mechanical environment. Some of these parameters are discussed in following, and the parameter values are summarized in Table 4.

**Table 4:** Mechanical properties of different native and BNC bypass grafts

Scaffold type	Test direction	Ultimate tensile strength/tensile strength [MPa]	Strain/elongation at failure [%]	Elastic/ Young’s module [MPa]	Burst strength [mmHg]	Suture retention [*N*]	Reference
**Saphenous vein**	Longitudinal	6.3	83	23.7	Not available	≈1.8	[154, 155].
		13	17	130			
	Circumferential	1.8	243	4.2	1680-3900		
		3	11	43	Not available		
		4	180	2.25	1250		
**Mammary artery**	Circumferential	Ultimate stress: 0.726 ± 0.352	0.314 ± 0.059	3.108 ± 1.652	Not available	1.38 ± 0.50	[155, 156]
**Left internal mammary artery**	Longitudinal	4.3	59	16.8	Not available		[154]
	Circumferential	4.1	134	8	2000		
**Bacterial nanocellulose /polyurethane**	Axial	1.22 ± 0.03	92.55 ± 29.64	1.51 ± 0.27	2156.25 ± 122.95	2.40 ± 0.46	[121]
	Radial	0.73 ± 0.08	293.35 ± 58.28				
**Bacterial nanocellulose/embedded cobalt–chromium mesh**	Longitudinal	0.052	Not available	Not available	Not available	8.83 ± 1.47	[157]
	Circumferential	0.018					
**Bacterial nanocellulose/polyvinyl alcohol**	Axial	0.17	39.1	≈ 0.8	352,53	≈ 0.39	[101, 144]
**Bacterial nanocellulose**	Longitudinal	1.45	14	Not available	Not available	Not available	[38]
		1.6	17				
		2.55	12				

Developing cardiovascular bypass grafts that closely mimic the mechanical properties of native arteries is important to ensure long-term patency of the grafts [112].To support this goal, a variety of testing methods are established to evaluate the mechanical performance of scaffolds applied in cardiovascular tissue engineering. Specifically, the mechanical testing of cardiovascular scaffolds such as tubular vascular grafts and patches is typically carried out according to the ANSI/ISO 7198:2016 standard, which serves as a complement to the more general ISO 14630:2012 standard [113].

The methods recommended for evaluating the mechanical properties of materials used in cardiovascular grafts include circumferential and longitudinal tensile strength, dynamic radial compliance test, pressurized burst strength, suture retention strength and kink diameter/radius [113, 114]. These tests collectively aim to capture both the load-bearing capacity and the deformation behavior of vascular grafts under physiological and supraphysiological conditions. In this section, the most applied methods to evaluate the mechanical properties of BNC are described.

### Tensile and ring tensile test

According to the ANSI/ISO-7198: 2016 standards the tensile test can be performed by applying the tensile deformation at a constant strain rate between 50–200 mm/min until rupture or a certain strain value. Some studies report differing strain rates from the ISO recommended strain rate [38, 115, 116]. Of note, due to the sample geometry, the performance and data analysis vary. The tests are performed in dried or hydrated state (in a phosphate-buffered saline (PBS)), at room temperature or at 37 °C. In addition, the tests are carried out in circumferential and/or longitudinal directions. For the ring-shaped sample like BNC, the test is adapted by using hooks or pins to hold the sample, depending on the sample size. According to the ISO 7198 the length of the ring samples should not be less than the nominal relaxed internal diameter [113].

After applying the sample on the tensile machine, stress-strain curves are generated. These curves typically exhibit a characteristic J-shaped behavior for vascular tissues, reflecting the progressive recruitment of collagen fibers under increasing strain. The stress and the strain rate are calculated using two equations:

$$\sigma =\frac{F}{{A}_{0{cs}}}$$

$$\varepsilon =\frac{l-{l}_{0}}{{l}_{0}}=\,\frac{\Delta l}{{l}_{0}}$$

Here, σ is stress [Pa], F is the force [N], A_0cs_ is the initial cross-section area in relaxed phase [m^2^], ε is the strain [-], *l* distance between the plates or pins (in case of ring tensile test) [m], and *l*_0_ is the initial distance [m]. With regard to any ring-shaped samples (such as BNC), the initial cross-section area is calculated using the equation:

$${A}_{0{cs}}=2{Wt}$$

**Fig. 3:**
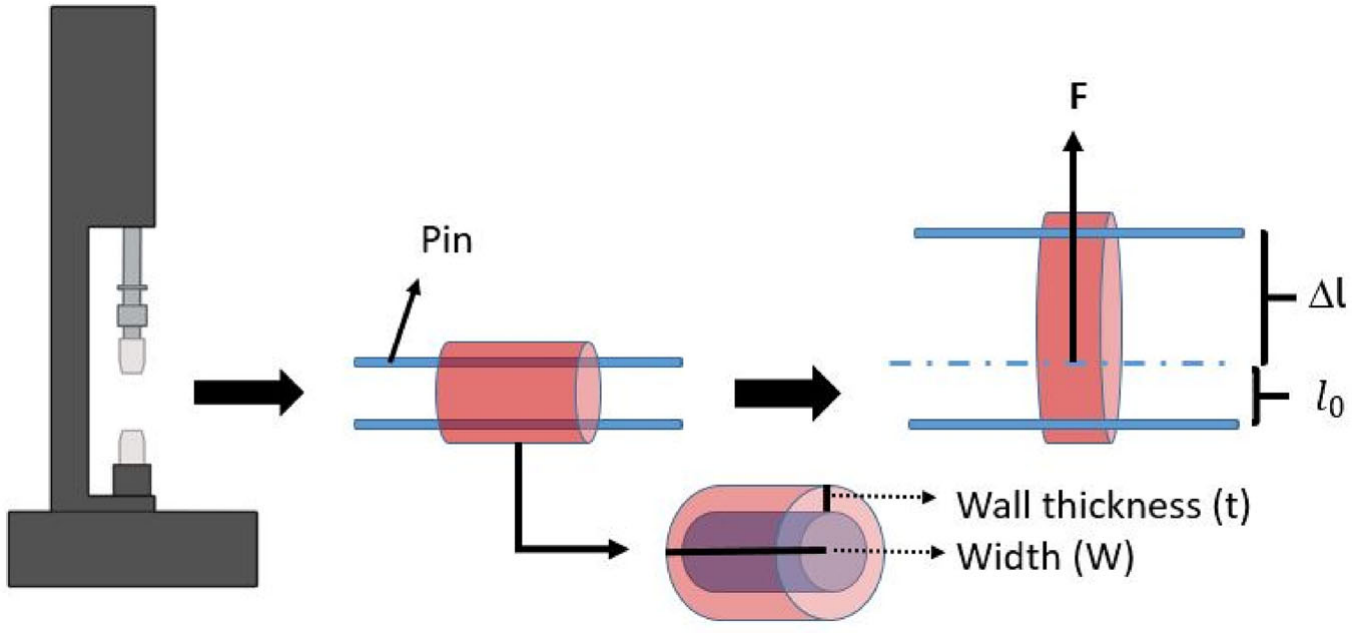
Scheme of ring tensile test in circumferential direction. F: force [N], l0: the initial distance between the pins [m], ∆l: elongation [m], t: wall thickness [m] and W: width [m]. Created with BioRender.com

Here, W is the sample width, and t is the sample thickness (Fig. 3). Additionally, the elastic modulus (Young’s modulus) can be calculated by the linear slope of the stress-strain curves in longitudinal and circumferential conditions. Due to the challenges in measuring the exact wall thickness of soft prosthesis and potential errors, some studies reported using the tensile force instead of the true stress [113, 116]. This highlights a common limitation in tensile tests, where variations in sample geometry and thickness can significantly affect the calculated mechanical properties.

### Tensile stress-relaxation (TSR)

TSR refers to a gradual decrease in stress under constant strain over time. This test describes the viscoelastic behavior of the material and provides insights into the mechanical behavior of a material (e.g., BNC-based vascular prosthesis). In this test, the specimen is deformed to a fixed strain, and the load required to maintain the deformation is measured over time. This behavior (a gradual decrease in tension with constant elongation over time) can be seen in viscoelastic materials due to the rearrangement of the molecules within the construct. The initial elastic modulus represents immediate response of the given material to strain, while the relaxed elastic modulus corresponds to the stress level at the end of the relaxation period [113, 117, 118].

The most common model applied to analyze the stress relaxation behavior of soft tissues is the Maxwell-Wiechert model which is comprised of a linear spring, characterized by elastic modulus Ei (the spring constant of the *i*-th spring) and multiple dashpots, each defined by a viscosity ƞ_i_ (the viscosity of the *i*-th dashpot (viscous element) in this model via the equation:

$$\sigma \left(t\right)={\varepsilon }_{0}.\,{E}_{E}+{\varepsilon }_{0}.{E}_{1}.\exp \left(-\frac{t}{{\tau }_{1}}\right)+{\varepsilon }_{0}.\,{E}_{2}.\exp \left(-\frac{t}{{\tau }_{2}}\right)+\ldots +{\varepsilon }_{0}.{E}_{n}.\exp \left(-\frac{t}{{\tau }_{n}}\right)\,$$

The calculation of stress–strain curve, direction of load and sample geometry are similar to tensile and ring tensile testing [113].

### Burst pressure testing

The burst pressure test evaluates the mechanical properties of vascular prostheses, by simulating the force exerted by blood pressure. This method directly assesses the burst pressure of tubular constructs by infusing either liquid or gas, at a constant rate inside a hermetically sealed sample until it bursts. The pressure is monitored and recorded in a data acquisition program. Burst pressure is defined as the maximum internal pressure that the graft can resist before structural failure occurs, and is therefore a direct measure of its mechanical integrity under physiological stress conditions. The ANSI/ISO 7198:2016 recommends the application of a non-permeable, elastic sleeve with higher burst pressure on the luminal side of the sample, depending on the porosity and permeability. By infusing the solution under constant rate, the diameter of the sample is measured by a laser micrometer until rupture and pressure diameter curves are generated [113]. According to the ANSI/ISO 7198:2016, the burst pressure test could also be performed with gas. Gas offers precise pressure control, faster response and the ability to detect microleaks, make it more effective in low-pressure or small-volume systems like tubular soft-tissue constructs like BNC [113, 119]. Using gas is considered to be simpler, easier and faster in comparison to the usage of liquids. On the other hand, using gas has several limitations, like potential of different pressure responses compared to liquids due to gas compressibility, and gas testing does not replicate precise physiological conditions as effectively as fluid-based methods [102, 113, 120].

Several safe-made systems for measuring burst pressure have been described. Li, Geli, et al. [121] evaluated a self-made device to measure the burst pressure of elastic bacterial nanocellulose/polyurethane small caliber as artificial blood vessel, where the one side of the tubular sample with a length of 4 cm is connected to a syringe and the other side to a pressure gauge using water at a speed of 0.01 MPa s^−1^ (75 mm Hg s^−1^), the pressure was recorded until rupture [121, 122].

Laterreur et al. used a direct method by pressurizing a tubular construct by pressurizing the construct with phosphate buffered saline (PBS), using a custom-made system. The system includes a chamber filed with PBS, a syringe pump, a pressure transducer, and a digital micrometer to monitor pressure and diameter until failure. Both sides of the tubular construct were cannulated and secured in position (in chamber) by silk sutures to prevent leakage. One end was connected to the syringe pump, while the other was sealed. Before testing the construct was pre-conditioned by cycling the internal pressure between 70–130 mmHg. The pressure was increased afterwards until burst. The results of the direct method were then compared with estimated by a ring tensile test [123].

### Suture retention test

The suture retention test is especially popular in cardiovascular field because of the use of sutures and staples to connect the implants to the native tissue. According to the ANSI/ISO 7198:2016 this test aims to determine the force (newton) that is required to pull a suture through material respectively the wall of a vascular prosthesis. This parameter is especially relevant from a surgical perspective, as insufficient suture strength can lead to anastomotic failure or leaks. Briefly, the suture is inserted 2 mm from the end of the stretched sample, loaded to a uniaxial tensile machine at 0° (parallel), 45° and 90° (perpendicular) to the longitudinal axis of the graft, representing different type of surgical anastomoses, and it is pulled at the rate of 50 mm/min to 200 mm/min until it tears out. The maximal force is measured and used as suture retention strength [102, 113, 124, 125]. Bao, Luhan, et al. evaluated suture retention strength of an air-dried bacterial nanocellulose conduit (BNC hydrogel-like conduit (BNC-Gel) and air-dried BNC (BNC-Dry)) with a length of 5 cm using the universal material testing machine (H5K, Hounsfield, UK). The sample was sutured 2 mm from the edge of sample with a 5–0 Dacron or 10-0 nylon suture. The tensile force was then applied at a rate of 50 mm/min until failure. It has been reported that the suture retention strength were 0.35 and 0.34 N for BNC-Gel and 0.42 and 0.63 N for BNC-Dry comparable to the suture strength of human tissue-engineered vessel (0.46 N) [126]. Table 4 shows the mechanical properties of native and some BNC-based scaffold.

## Application of BNC grafts in cardiovascular research

Selection of an appropriate artificial vascular graft material poses a great challenge in cardiovascular tissue engineering. Traditionally, autologous materials are the most commonly used bypass grafts in cardiovascular surgery, as they minimize the risk of immunological reactions and thrombosis upon implantation. The use of arterial grafts has shown better long-term results, with more than 90% patency for almost 10 years [127]. As explained earlier concerning unavailability of autologous vessels due to patient conditions such as varicose veins or in older individuals, natural grafts are not always a promising option [7, 128, 129]. To address this specific challenge, various studies have been carried out to identify alternative materials for cardiovascular tissue-engineered grafts. Synthetic materials like poly-L-lactide (PLLA), poly-ɛ-caprolactone (PCL), polyglycolide (PGA), polytetrafluoroethylene (ePTFE), and polyethylene terephthalate (PET, Dacron) grafts have been developed, but these attempts have given only partial success [109, 130, 131]. In line with this, the usage of ePTFE and Dacron grafts have failed to deliver acceptable patency rates, with only 60% in 1 year and 14% in 3 years [8, 132, 133]. The main reason for these unsuccessful attempts is the lack of endothelialization, which leads to significant increases in graft occlusion rates. Therefore, selecting an optimal material with a high potential for endothelialization within the implant is crucial for increasing patency rates for artificial bypass grafts [127].

Though BNC may be non-adherent to endothelial cells due to lack of extracellular matrix structures, there are possibilities for its surface modifications to enhance endothelialization, which is crucial for an artificial small-caliber vascular graft. Though BNC has often been described as a biodegradable material, its degradation in the human body remains limited. Humans lack cellulase enzymes capable of cleaving the β-1,4-glycosidic bonds of cellulose, which explains the limited degradation of BNC by enzymatic processes in vivo. Furthermore, comprehensive data on the hydrolytic degradation of BNC under physiological conditions remain limited, and the long-term outcome of implanted BNC is not yet fully understood [134]. While surface modification strategies have been shown to improve the endothelialization and tissue integration of BNC scaffolds, similar improvements have also been reported for other vascular graft biomaterials such as PLLA [135], PCL [136], and ePTFE [137, 138]. Table 5 represents different materials applied in cardiovascular tissue engineering compared to commercially available grafts, indicating that bacterial nanocellulose demonstrates high potential as a small-caliber vascular graft compared to the other materials.

**Table 5:** Materials applied in cardiovascular tissue engineering

Material	FDA	Role in Vascular Grafts	Advantages	Limitations	Ref.
**Bacterial Nanocellulose (BNC)**	No	cellulose based scaffold with limited biodegradability in human body, surface modification may enhance endothelialization and tissue integration	High biocompatibility, adjustable mechanical properties, improved burst pressure, promising scaffold as small caliber graft ( < 6 mm)	Limited long-term clinical data, reproducibility, needs modification to improve endothelialization	[158, 159]
**Poly-L-lactide (PLLA)**	No	Slow biodegradable scaffold	High tensile strength, supports cell attachment and tissue regeneration	A slow degradation could lead to chronic inflammation and a risk of thrombosis.	[160, 161]
**Poly-ɛ-caprolactone (PCL)**	No	Slow biodegradable scaffold	Excellent mechanical properties, support cell attachment, low degradation rate,	extreme slow degradation could lead to chronic inflammation and a risk of thrombosis.	[162]
**Polyglycolide (PGA)**	No	Fast biodegradable scaffold	Fast degradation supports tissue regeneration, improved mechanical strength	Rapid degradation may lead to insufficient mechanical support, potential for thrombosis, not ideal as small caliber vascular graft	[163]
**Expanded Polytetrafluoroethylene (ePTFE)**	Yes	synthetic scaffold	High patency rates in large-caliber vessels, resistant to thrombosis	Poor performance in small-caliber vessels ( < 6 mm), low long-term patency rates	[164, 165]
**Polyethylene Terephthalate (PET, Dacron)**	Yes	synthetic scaffold	High patency rates in large-caliber vessels, resistant to thrombosis	Poor performance in small-caliber vessels ( < 6 mm), low long-term patency rates	[166, 167]
**Symvess™ (Humacyte, 2024)**	Yes	Arterial replacement for trauma	No donor tissue required, ready-to-use, potential for universal compatibility	Limited to trauma applications, not approved for small-caliber vascular applications	[168, 169]

In this context, Li, G. et al. [121] developed a BNC/polyurethane (PU) conduit as a small caliber vascular graft via static fermentation method, using a fermentation medium consisting of fructose as a carbon source, tryptone and yeast extract inoculated with *Komagataeibacter xylinus*, DHU-ATCC-1. After the post-treatment step, a PU solution was added to the dried BNC tube for 12 h to allow the PU solution to penetrate the BNC wall. The results showed enhanced mechanical properties, cytocompatibility and hemocompatibility in vitro and rapid in situ endothelialization in vivo. In particular, in terms of cytocompatibility, they evaluated the initial cell adhesion by counting the number of cells on each surface 24 h after seeding, resulting in highest attachment on PU and lowest on BNC. The low rate on BNC is likely due to the strong hydrophilicity of BNC, which hindered early adhesion. Moreover, the cell proliferation was monitored over 1, 3 and 5 days. The result showed that while PU supported early attachment, BNC promoted the highest endothelial cell growth at day 3, which suggesting the influence of surface properties on long-term biocompatibility. The 3D nanofibrous structure combined with moderate hydrophilicity of the BNC/PU scaffold seemed to support continuous proliferation. However, there was a drawback as the grafts were characterized by an excessive wall thickness that neither matched with autologous blood vessels nor obtained the compliance required for human coronary arteries.

Regarding the surface characteristic of BNC on hemocompatibility and cytocompatibility, a study was done to investigate the mechanisms involved in the biocompatibility of both sides of the BNC, since BNC membrane is generally formed with heterogeneous surfaces, having a smooth upper surface and a rough lower surface [99]. For this purpose, a BNC membrane was prepared by inoculating 1.25% (w/v) *Komagataeibacter xylinus* in 100 ml culture medium consisting of glucose as carbon source, tryptone and yeast powder at a pH of 5–5.2 and incubated under static conditions at 30 °C for 5 days. After the post-treatment process, the BNC membrane was sterilized in an autoclaved. The morphology and the effects of surface properties on protein absorption, hemocompatibility and cytocompatibility were then investigated. The results showed that both sides of the BNC membrane are non-hemolytic and meet the ASTM F756 standard (< 2%) [99, 139]. The cell responses were also investigated by analyzing the gene expression of human umbilical vein endothelial cells (HUVECs) on the BNC membrane surfaces with upper surface up or lower surface up in a 24-well plate containing 500 μl culture medium. The results showed that HUVECs had higher cluster of differentiation 31 (CD31) expression and endothelial nitric oxide synthase (eNOS) genes with 1.15-fold and 1.12-fold increases, respectively, compared with cells on the upper surface of BNC. Evaluation also showed lower expression of intercellular adhesion molecule-1 (ICAM-1) genes (0.93-fold decrease) on the lower surface of BNC membrane. This means that the upper BNC surface shows greater hemocompatibility, while the lower BNC surface shows better cytocompatibility.

To summarize, the different bioactivity of the two surfaces could prevent the optimizing of hemocompatibility and cytocompatibility at the same time. To achieve balanced hemocompatibility and cytocompatibility in order to improve endothelialization in vivo, targeted surface modification, in particular adjustment of roughness or coating with bioactive substances, could be performed.

The biomechanical properties, cell density, homogeneity, quality of bacterial nanocellulose and surface characteristic of BNC are another key aspect, enhancing the endothelialization by affecting the characteristics, hemocompatibility and cytocompatibility of BNC as a small caliber vascular graft. Thus, several studies are performed in order to enhance these properties [121, 140, 141].

### Application of BNC scaffolds as vascular prosthesis

Recently, several studies have been performed to implant BNC scaffolds in animal models in order to investigate its properties and performance as a vascular prosthesis. For instance, Hu, G., et al. implanted a mercerized BNC/ polyvinyl alcohol (PVA)-12.5 (MBP-12.5) conduit in an abdominal aorta in Sprague-Dawley (SD) rat model. Here, the abdominal aorta was clamped, transected, and an MBP-12.5 conduit was implanted. After implantation, Doppler sonographic assessment was performed at 1, 4, 8, 12, 16, 20, and 32 weeks. After 32-week, implants were harvested and analyzed with Masson’s trichrome staining, immunofluorescent staining (α-SMA and CD-31) and H&E staining. Histological analysis indicated extracellular matrix deposition and collagen formation around the implanted prosthesis without significant inflammatory response. Human umbilical vein endothelial cells (HUVECs) were primarily used to evaluate the in vitro cytocompatibility of the scaffold. Masson’s trichrome staining showed increasing the collagen during the experiment. Moreover, a few positive inflammatory cells were located between the sample and the tissue in weeks 1 and 2 and decreased from week 2 to week 4. Doppler sonographic results also showed normal blood flow through the MBP-12.5 prosthesis. However, this prosthesis has some limitations, such as poor mechanical properties comparing of native blood vessels (Table 4) [101].

Leitao, A.F., et al. also implanted tubular BNC with the lengh of 3–4 cm anastomosed with continuous suture with 6/0 monofilament polypropylene sutures in the left hind limb in a homolateral-femoralartery in female domestic pig (Sus scrofa domesticus) for 2 months. Doppler sonographic results showed a confirmed patency after one month. Histological analysis represented a structure with cellular adhesion (believed to be endothelial or progenitor endothelial cells that migrated from the adjoining femoralartery) on the luminal and adventitial surface of BNC with no significant inflammation. However, after two months the grafts were found occluded by thrombi, consisting a large population of fibroblast and macrophage and a small population of lymphocytes. These results were observed during the early stages, and further investigation is needed to assess long-term patency [142].

Another study performed by Scherner et al. investigated the in vivo functional performance and pro-inflammatory potential of BNC prostheses implanted into the carotid arteries of 10 female sheep (Texel sheep) over a period of 3 months. Here, about 100 mm of the left common carotid artery was resected, and a BNC graft was anastomosed end-to-end using ten interrupted 6–0 monofilament nylon sutures. Doppler ultrasound was performed after 4, 8, and 12 weeks to measure blood flow velocity.

The results showed a patency rate of 50% after 12 weeks. Sonography analysis showed a physiological functional performance of the BNC grafts without diameter reduction or non-laminar flow profiles. The blood flow velocity of the patent BNC grafts showed no significant difference compared to the native contralateral common carotid artery (35.9 ± 3.8 m/s vs. 39.2 ± 4.5 m/s). Moreover, the BNC grafts showed no macroscopic signs of inflammation and did not reveal giant cells or other inflammatory reactions, such as a distinct number of small cells, in histological analysis within and around the grafts [143].

Taken together, the main limitations of BNC grafts in cardiovascular applications include their insufficient early endothelialization, poor elasticity and compliance mismatch compared to native vessels, excessive wall thickness and limited long-term patency and reproducibility due to challenges in achieving uniform, mechanically stable small-caliber constructs. These findings provocate further for improving the surface properties, production quality and biocompatibility of BNC for utilizing it as a small caliber vascular graft. For cardiovascular applications, the fabrication of homogenous and uniform BNC graft must address characteristics like a reduced wall thickness of max. 1 mm, an inner diameter of 3–4 mm and improved mechanical properties, such as elastic modulus between 2 and 4 Mpa.

### Current limitations and challenges for clinical translation

Though BNC exhibits promising properties for vascular graft applications, several critical limitations still prevent its successful clinical application. One of the most significant challenges involves poor endothelialization. Native BNC lacks cell adhesion properties due to lack of adhesion proteins, extracellular matrix components necessary for rapid endothelial cell attachment and formation of a confluent antithrombotic layer. Delayed or limited endothelialization increases the risk of thrombosis and graft occlusion, especially in small-diameter applications. In various studies investigating tissue-engineered vascular grafts employed surface modifications on BNC templates with synthetic polymers to overcome its limited ability to support endothelial cell adhesion and proliferation [101, 121, 144]. While some coatings showed promising results, the coating’s impact on hemocompatibility and mechanical properties of the grafts was not thoroughly tested, limiting clinical translation.

In addition, hemocompatibility has not been fully established, as most studies have primarily focused on hemolysis testing, while other key factors influencing thrombotic behavior, such as platelet activation, complement activation, coagulation cascade initiation, and leukocyte interactions, have not been systematically or comprehensively evaluated. Although several studies report low hemolysis rates, comprehensive evaluation of platelet activation, complement activation and leukocyte response remains limited. In addition, hemolysis testing alone does not adequately predict thrombotic performance in vivo. Thrombosis is a multifactorial process involving platelet activation, coagulation cascade initiation, complement activation, and leukocyte interactions. Recent translational reviews emphasize that comprehensive hemocompatibility assessment—particularly under dynamic flow conditions—is essential for vascular graft evaluation [99, 102, 145, 146].

Mechanical compatibility with natural arteries is another big challenge. While BNC exhibits desirable tensile strength, parameters such as wall thickness, radial compliance and long-term mechanical stability need to closely match those of natural vessels to avoid hemodynamic disturbances and intimal hyperplasia [113, 147, 148]. Producing homogeneous grafts with small diameters, controlled wall thickness, and reproducible mechanical properties remains a technical challenge.

## Conclusion and future perspectives

As a conclusion, the selection of BNC-producing species and an optimal bacterial count, along with control on different production parameters such as temperature, pH, oxygen content, nutritional supplements in the medium and surface modification could possibly pave way to produce a suitable BNC graft that closely resembles the properties of human coronary arteries. These parameters directly impact the yield, fiber morphology and crystallinity, which leads to changes in mechanical properties of BNC. However, further investigations are required to deeply understand these aspects and to gain a comprehensive understanding of structural, mechanical, and biological characteristics of a BNC graft. These properties include wall thickness of max. 1 mm and an inner diameter of 3–4 mm, enhanced surface roughness in order to better cell attachment and proliferation, improved mechanical properties (elastic modulus between 2–4 MPa), a burst pressure above 1000 mmHg and surface modification via various coatings. These properties could be achieved by modification of the surface that the bacteria grow on, increasing the number of bacteria by controlling the temperature, oxygen rate during BNC production and adding additive components such as ethanol, citric acid and extra carbon source like fructose in order to enhance the mechanical properties and burst pressure. Such studies will contribute to optimizing BNC for biomedical applications, particularly as bypass grafts in cardiovascular tissue engineering.
